# A systematic review of digital interventions for smoking cessation in patients with serious mental illness

**DOI:** 10.1017/S003329172300123X

**Published:** 2023-08

**Authors:** Luis Martinez Agulleiro, Bhagyashree Patil, Joseph Firth, Chelsea Sawyer, Benedikt L. Amann, Francina Fonseca, Marta Torrens, Victor Perez, Francisco Xavier Castellanos, John M. Kane, Daniel Guinart

**Affiliations:** 1 Department of Child and Adolescent Psychiatry, New York University Grossman School of Medicine, New York, NY, USA; 2 Department of Psychiatry, Maimonides Medical Center, Brooklyn, NY, USA; 3 Division of Psychology and Mental Health, The University of Manchester, Manchester Academic Health Science Centre, Manchester, UK, M13 9 PL; 4 Institute of Neuropsychiatry and Addictions (INAD), Parc de Salut Mar, Barcelona, Spain; 5 Hospital del Mar Medical Research Institute (IMIM), Barcelona, Spain; 6 Centro de Investigación Biomédica en Red de Salud Mental (CIBERSAM), Instituto Carlos III, Madrid, Spain; 7 Universitat Pompeu Fabra, Barcelona, Spain; 8 Universitat Autònoma de Barcelona, Bellaterra, Spain; 9 Universitat de Vic i Central de Catalunya, Vic, Spain; 10 Nathan Kline Institute for Psychiatric Research, Orangeburg, NY, USA; 11 Department of Psychiatry Research, The Zucker Hillside Hospital, Glen Oaks, NY, USA; 12 Institute of Behavioral Science, Feinstein Institutes for Medical Research, Manhasset, NY, USA; 13 The Donald and Barbara Zucker School of Medicine at Hofstra/Northwell, Manhasset, NY, USA

**Keywords:** bipolar disorder, depression, digital health, e-Health, schizophrenia, smoking cessation, tobacco

## Abstract

Tobacco smoking is highly prevalent among patients with serious mental illness (SMI), with known deleterious consequences. Smoking cessation is therefore a prioritary public health challenge in SMI. In recent years, several smoking cessation digital interventions have been developed for non-clinical populations. However, their impact in patients with SMI remains uncertain. We conducted a systematic review to describe and evaluate effectiveness, acceptability, adherence, usability and safety of digital interventions for smoking cessation in patients with SMI. PubMed/MEDLINE, EMBASE, CINAHL, Web of Science, PsychINFO and the Cochrane Tobacco Addiction Group Specialized Register were searched. Studies matching inclusion criteria were included and their information systematically extracted by independent investigators. Thirteen articles were included, which reported data on nine different digital interventions. Intervention theoretical approaches ranged from mobile contingency management to mindfulness. Outcome measures varied widely between studies. The highest abstinence rates were found for *mSMART MIND* (7-day point-prevalent abstinence: 16–40%). *Let's Talk About Quitting Smoking* reported greater acceptability ratings, although this was not evaluated with standardized measures. Regarding usability, *Learn to Quit* showed the highest System Usability Scale scores [mean (s.d.) 85.2 (15.5)]. Adverse events were rare and not systematically reported. Overall, the quality of the studies was fair to good. Digitally delivered health interventions for smoking cessation show promise for improving outcomes for patients with SMI, but lack of availability remains a concern. Larger trials with harmonized assessment measures are needed to generate more definitive evidence and specific recommendations.

## Introduction

Tobacco smoking kills more than 8 million people a year worldwide (World Health Organization, 2019), making it the third highest risk factor for attributable disability-adjusted life-years, surpassed only by child and maternal malnutrition and high systolic blood pressure (Murray et al., 2020). Total global costs of tobacco smoking are estimated around USD 1432 billion, which represents 1.8% of global annual GDP (Goodchild, Nargis, & Tursan d'Espaignet, 2018). In the US, tobacco is the leading cause of preventable death and disability (Center for Disease Control and Prevention, 2021), with direct and indirect medical costs exceeding USD 300 billion (US Department of Health Human Services, 2020; Xu, Bishop, Kennedy, Simpson, & Pechacek, 2015).

The excess premature mortality associated with smoking can be mitigated by smoking cessation (Doll, Peto, Boreham, & Sutherland, 2004), but treatments are underutilized, and most major clinical trials have excluded those with serious mental illness (SMI) (Cather, Pachas, Cieslak, & Evins, 2017). Smoking rates have declined significantly over time among individuals without mental illness, but changed only slightly among those with mental illness (Cook et al., 2014). More than half of individuals with schizophrenia spectrum disorders smoke cigarettes, a rate three times that of the general population (Sagud, Mihaljevic Peles, & Pivac, 2019; Šagud et al., 2018). Smoking prevalence is also particularly high among those with bipolar disorder (BD), who are two to three times more likely to have started smoking and are less likely to initiate and/or maintain smoking abstinence than individuals without psychiatric disorders (Heffner, Strawn, DelBello, Strakowski, & Anthenelli, 2011). Life expectancy for individuals with SMI is reduced compared with the general population (Plana-Ripoll et al., 2019), with tobacco accounting for a substantial portion of total deaths among patients with schizophrenia or BD (Callaghan et al., 2014).

Smoking cessation interventions would seem to be fundamental for improving life expectancy and quality of life in people with SMI (Doll et al., 2004; World Health Organization, 2018). However, mental health services are often unable to provide sufficient support for smoking cessation to patients (Bailey et al., 2019), and staff attitudes about lack of usefulness of these strategies hinder the achievement of tobacco abstinence (Malone, Harrison, & Daker-White, 2018). Furthermore, individuals with SMI appear to have specific mediators/moderators of smoking behavior and barriers to smoking cessation compared to the general population (Lum, Skelton, Wynne, & Bonevski, 2018; Moran, Betts, Ongur, & Janes, 2018; Slyepchenko, Brunoni, McIntyre, Quevedo, & Carvalho, 2016). Smoking cessation self-efficacy, or confidence in one's ability to quit, is a known predictor of cessation outcomes with lower cessation self-efficacy predicting higher rates of relapse (Clyde, Pipe, Els, Reid, & Tulloch, 2017). Smokers with psychotic disorders such as schizophrenia commonly report low cessation self-efficacy, along with low motivation to quit due to a desire to better cope with symptoms, particularly cognitive impairment and negative symptoms, as well as to reduce side effects of antipsychotic treatment (Kumari & Postma, 2005; Lum et al., 2018; Thornton et al., 2012). When schizophrenia patients attempt to quit, some reports find exacerbation of executive functioning deficits, which may contribute to high rates of smoking relapse (Tidey & Miller, 2015). In the case of BD, a substantial portion of patients report that they smoke to manage symptoms of their mental illness, and smoking is associated with greater severity of mood symptoms, comorbid psychiatric and addictive disorders, and suicidality (Tidey & Miller, 2015). Experimental evidence supports that nicotine can indeed influence symptoms in SMI (Featherstone & Siegel, 2015; Koukouli et al., 2017; Kumari & Postma, 2005; Novak et al., 2010; Sabe, Zhao, & Kaiser, 2020), as well as cognitive performance in schizophrenia, BD and major depressive disorder (MDD) (Barr et al., 2008; Caldirola et al., 2013; Hong et al., 2011; Jubelt et al., 2008; Levin, Wilson, Rose, & McEvoy, 1996; Smith, Singh, Infante, Khandat, & Kloos, 2002; Smucny, Wylie, Kronberg, Legget, & Tregellas, 2017). Other studies, however, limit the neurocognitive effects of nicotine only to schizophrenia (Morisano, Wing, Sacco, Arenovich, & George, 2013).

Pharmacologic treatments for smoking cessation in SMI include bupropion and varenicline, as well as nicotine replacement therapies when necessary (Peckham, Brabyn, Cook, Tew, & Gilbody, 2017). However, quit rates in SMI are low and relapse rates are higher than in the general population (Brunette et al., 2018b; Peckham et al., 2017). While psychosocial treatments like cognitive behavioral therapy, motivational interviewing, and contingency reinforcement have demonstrated short-term efficacy in some trials, other studies have shown no effects (Baker et al., 2006; Cather et al., 2017; Gelkopf et al., 2012; Gold et al., 2018; Lum et al., 2018; Williams et al., 2010). With the advance of digital technologies and eHealth, several smoking cessation digital interventions have been developed for use in non-psychiatric population, although evidence regarding their efficacy shows mixed results (Bricker, Watson, Mull, Sullivan, & Heffner, 2020; Chulasai, Chinwong, Chinwong, Hall, & Vientong, 2021; Garrison et al., 2020; Pbert et al., 2020; Peek et al., 2021). Although digital interventions would seem to be particularly relevant for patients with SMI (Sawyer, Hassan, Guinart, Martinez Agulleiro, & Firth, 2022), most major clinical trials have excluded such patients (Cather et al., 2017), and most interventions have not been assessed in properly powered clinical trials (Haskins, Lesperance, Gibbons, & Boudreaux, 2017), have not included behavior change techniques that have been shown to be effective in smoking cessation interventions (Ubhi et al., 2016) or have not been customized to match users' personal characteristics (Hoeppner et al., 2016). A recent narrative article has explored the feasibility of smoking cessation apps in people with schizophrenia (Sawyer et al., 2022), and indicated that these interventions might represent a realistic alternative for the treatment of nicotine use in patients with SMI. However, due to its narrative nature, information on effectiveness, usability, and other relevant outcomes was scarce and not systematically assessed.

Thus, the purpose of this review was to systematically describe and evaluate the characteristics of digital interventions for smoking cessation validated in patients with SMI, namely schizophrenia-spectrum disorder (SSD), BD, and MDD. We aimed to describe and evaluate (1) effectiveness, (2) acceptability and adherence, (3) usability, and (4) safety of these interventions.

## Methods

This systematic review was conducted according to Preferred Reporting Items for Systematic Reviews and Meta-Analysis (PRISMA) guidelines (Moher, Liberati, Tetzlaff, Altman, & Group, 2009). The review protocol for this review was registered in PROSPERO (CRD42021262481).

### Literature search

We conducted a search of the electronic databases PubMed/MEDLINE, EMBASE, CINAHL, Web of Science, Cochrane Tobacco Addiction Group Specialized Register, and PsycINFO from database inception until 31 March 2022. Reference lists of eligible studies were screened, but no other sources (including unpublished studies) were sought. No restrictions on language were applied. Published, full-text peer-reviewed articles reporting the effectiveness, acceptability, adherence, usability and/or safety of digital interventions for smoking cessation in patients with SSD, BD, and/or MDD were included. Articles were excluded if: (1) the study population was composed of patients with a primary diagnosis different from SSD, BD and/or MDD; (2) the intervention was exclusively non-digital; (3) the intervention was not for smoking cessation; and (4) the paper was an abstract, systematic review, protocol, or poster communication. The search string was designed to include any study reporting digital interventions for smoking cessation in patients with SMI, as follows: (digital OR app OR web OR ehealth OR mhealth OR smartphone) AND ((smoking OR tobacco) AND (cessation OR abstinence)) AND (severe mental illness OR serious mental illness OR SMI OR psychosis OR schizophrenia OR schizoaffective OR bipolar OR mania OR major depressive disorder OR major depression OR depression).

### Study selection and data extraction

After initial search and removal of duplicates, each study was independently screened for eligibility by two reviewers (BP, LMA). If a study was deemed relevant by at least one reviewer, or in case of doubt, the full-text article was retrieved and examined in depth for eligibility. Differences in search and selection results were discussed and when consensus was not reached, a third reviewer (DG) was consulted.

Data extraction was performed independently by two reviewers (BP, LMA) using a template developed for this systematic review including the following characteristics and data: (1) article design and characteristics: first author, year, country, study design, primary and secondary outcomes, comparators; (2) characteristics of the digital intervention: name, type (smartphone app, web), device (smartphone, cellphone, laptop, computer), cost, theoretical approach, duration; (3) sample characteristics: eligibility criteria, sample size, diagnosis, age, sex, race, illness duration, setting (inpatient and/or outpatient), symptom severity, pattern of tobacco use and severity of nicotine dependence; and (4) results of the intervention: effectiveness, acceptability, adherence, usability, and safety.

Data on effectiveness of the intervention were extracted according to study-defined criteria as any smoking reduction and/or abstinence/cessation, measured either as self-report, structured/semi-structured interviews, or expired carbon monoxide (CO). Acceptability, adherence and usability were extracted using the quantitative instruments reported in the study. Additionally, qualitative results on acceptability and usability were extracted and summarized from available articles. Safety of the intervention was extracted if the publication reported the onset of adverse events (AEs) during the trial.

Study quality was evaluated using the National Heart, Lung, and Blood Institute (NHLBI) Study Quality Assessment Tools for Controlled Interventions and Before-After (Pre-Post) Studies With No Control Group (National Institutes of Health, 2021). As most digital interventions were not accessible online to be fully evaluated with the *Adapted Mobile App Rating Scale* (A-MARS) (Stoyanov et al., 2015), we used author-reported information whenever possible (see online Supplementary Table S3). For the two interventions that were available (*QuitGuide* and *quitStart*), ratings reflect the assessment made independently and agreed upon by the investigators (BP, LMA) while using the apps.

## Results

**Figure 1.**
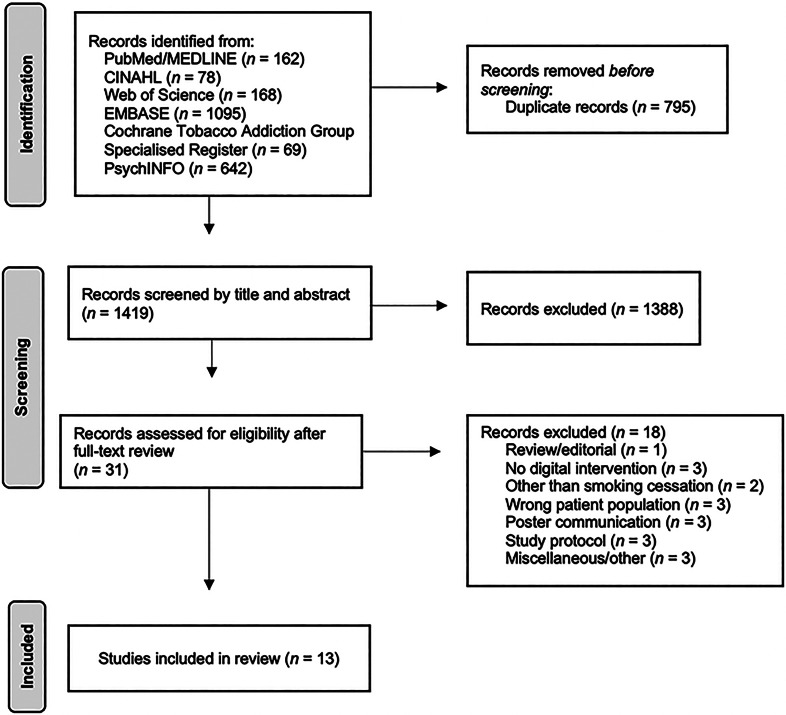
PRISMA flowchart of the review process.

The search retrieved 2214 articles. After duplicates were removed, 1419 unique studies were screened and 1388 were excluded. A full-text review of 31 articles was conducted, and finally 13 articles were included in this review. The inter-rater agreement was 98.5% [95% CI (97.6–99.0)]. The study selection process is presented as a PRISMA flow diagram in Fig. 1. A detailed summary of the characteristics of the included articles can be found in Table 1.

**Table 1.** Characteristics of the included studies

Study						Participants				Intervention		Quality
Author, year (country)	Study design	Primary outcome	Secondary outcomes	Diagnosis (*n*)	Sample size (*n*)	Sex; age [mean (s.d.)]; race	Setting	FTND [mean (s.d.)]	Cigarettes per day; years smoking [mean (s.d.)]	Name of the intervention	Comparator	NHLBI SQAT
(Brunette et al., 2011 (US))	Quasi-experimental (pilot study)	Use of smoking cessation treatment	–	SZ = 28, non-SMI = 13	Intervention = 21, control = 20	NR	Outpatient	NR	NR	Let's Talk About Smoking	None	Good
(Brunette et al., 2018a (US))	RCT (pilot study)	Use of smoking cessation treatment, quit attempts	Abstinence	SZ = 35	Intervention = 30, active control = 28, inactive control = 23	NR	Outpatient	NR	NR	Let's Talk About Smoking	Computerized education, none	Fair
(Brunette et al., 2019 (US))	Pre-post study (pilot study)	Feasibility, efficacy	–	SZ/SZ-AFF = 12, BD = 3, MDD = 3	20	NR	Outpatient	NR	NR	Let's Talk About Quitting Smoking	None	Good
(Brunette et al., 2020 (US))	RCT	Use of smoking cessation treatment, quit attempts	Abstinence, satisfaction, usability, likeability	SZ = 162	Intervention = 78, control = 84	66.7% male; 45.9 (11.3); 53.1% AA	Outpatient	5.2 (2)	14.6 (10.5); NR	Let's Talk About Smoking	Computerized education	Good
(Gowarty et al., 2021 (US))	Prospective	Usability, acceptability	–	SZ = 7	17	86% male; 30 (3.6); 86% Caucasian	Outpatient	5.1 (2.1)	18 (8.5); NR	QuitGuide, quitStart	None	NA
(Heffner et al., 2020 (US))	RCT (pilot study)	Feasibility, satisfaction, utilization, acceptability, smoking cessation outcomes	–	BD = 51	Intervention = 25, control = 26	55% male; 49 (10.8); NR	Outpatient	6.2 (2.1)	19.1 (8.2); NR	WebQuit Plus	Smokefree.gov	Good
(Medenblik et al., 2020 (US))	RCT (pilot study)	Acceptability, feasibility, smoking cessation knowledge	–	SZ = 15, SZ-AFF = 18, PNOS = 1	Intervention = 21, control = 13	80% male; 48.2 (9.9); 62% AA	Outpatient	5.9 (2.3)	17.6 (11.7); NR	iCOMMIT	Same program without mCM	Good
(Minami et al., 2018 (US))	Open-label, non-randomized, feasibility study (pilot study)	Feasibility, acceptability	–	MDD = 8	8	0% male; 55.3 (4.6); 50% AA	Outpatient	5.2 (1.9)	12.8 (6.9); NR	No name (mSMART MIND/SMI-CM)	Enhanced standard treatment with non-contingent CM	Fair
(Minami et al., 2022 (US))	RCT (pilot study)	Efficacy	–	BD = 24; MDD = 25	Intervention = 25, control = 24	24.5% male; 48.8 (11.6); Latinx 63.3%	Outpatient	4.8 (1.7)	10.5 (4.8); 29.4 (12.6)	No name (mSMART MIND/SMI-CM)	Enhanced standard treatment with non-contingent CM	Fair
(Vilardaga et al., 2016 (US))	Cross-sectional	User experience	–	SZ = 2; BD = 1; MDD = 1, SZ-AFF = 1	5	100% male; 51.2 (4.3); 20% Caucasian	Outpatient		NR; 28.8 (11.1)	QuitPal	None	NA
(Vilardaga et al., 2019 (US))	Case study, cross-over AB design without repeated measures	Usability, user experience, user engagement	–	SZ = 1; Mood with psychosis = 3; MDD = 1; PNOS = 2	7	42.9% male; 45 (9.5); 71.4% Caucasian	Outpatient	5	NR; 29	Learn to Quit	QuitGuide	NA
(Vilardaga et al., 2020 (US))	RCT (pilot study)	Feasibility, acceptability, efficacy	Smoking reduction, quit attempts	SZ/SZ-AFF = 15; BD = 30, MDD = 17	Intervention = 33, control = 29	40.3% male; 45.9 (11.1); 51.6% Caucasian	Outpatient	5.0 (2.5)	17.7 (12.1); 26.2 (12.2)	Learn to Quit	QuitGuide	Good
(Wilson et al., 2019 (US))	Successive cohort	Tailoring of the intervention	–	SZ = 7, SZ-AFF = 5, PNOS = 1	13	61.5% male; 47.8 (11); 76.9% AA	Outpatient	4.9 (1.8)	15 (6.6); 28.2 (13.7)	iCOMMIT	None	Fair

AA, African American; BD, bipolar disorder; CM, contingency management; FTDN, Fagerström Test for Nicotine Dependence; MDD, major depressive disorder; NA, not applicable; NHLBI SQAT, National Heart, Lung, and Blood Institute Study Quality Assessment Tools; NR; not reported; PNOS, psychosis not otherwise specified; RCT, randomized controlled trial; SMI, serious mental illness; SZ, schizophrenia; SZ-AFF, schizoaffective disorder.

The 13 included articles covered 9 different digital interventions. All included articles were published after 2011 and pertain to five distinct research groups. The number of articles studying each digital intervention ranged from 1 to 3. Six articles used controlled trial methods to study the digital intervention, while the rest used quasi-experimental and prospective designs. All samples were of outpatients with sample size ranging from *n* = 5 (Vilardaga et al., 2016) to *n* = 162 (Brunette et al., 2020). SSD was the most common diagnosis, followed by BD and MDD (Table 1).

### General characteristics of the included digital interventions

Nine unique digital interventions for smoking cessation were identified in this systematic review. At the time this review was conducted, only two (*QuitGuide* and *quitStart*) were commercially available and readily accessible via App Store and Google Play, all of them in English with no alternative language option. The remaining seven interventions were not accessible for evaluation and data were obtained from associated publications or directly from the authors whenever possible (See Table 2 for detailed description of the interventions). Briefly, six interventions were delivered as smartphone-based apps, and three were computer-based. The theoretical foundations of the digital interventions range from acceptance and commitment therapy (ACT) (Hayes, Strosahl, & Wilson, 2011) to theory of planned behavior (Ajzen, 1991), mindfulness (Quaglia, Brown, Lindsay, Creswell, & Goodman, 2015), and contingency management (CM) (Petry, 2011). The specific interventions were as follows:

1. *iCOMMIT* is a behavioral therapy-based smartphone-based app, which includes mobile CM (mCM). Additionally, the full intervention includes five cognitive-behavioral counseling sessions with a therapist. For the digital part of the intervention, participants are asked to self-record a video of themselves blowing into a handheld CO monitor to confirm smoking abstinence and upload it onto the secure website linked to the app. The website then analyses the CO monitor reading in the video and translates that information to deliver financial reinforcement through the app (Medenblik et al., 2020).
2. *Learn To Quit* is a smartphone-based app based on ACT and specifically developed for patients with SMI following a user-centered design (Vilardaga et al., 2018). Notably, it is the only included intervention who was developed in co-production with patients with SMI. It focuses on three processes of change: creating awareness of experienced smoking urges, openness to that experience, and committing to quitting smoking through specific values. *Learn To Quit* uses simple screens and large buttons, gamification and prioritizing visual engagement among other features to minimize usability challenges in people with SMI. It also implements a tracking option for daily ecological momentary assessments (EMA) of mood, number of cigarettes smoked, tobacco craving, and nicotine replacement therapy (NRT) use (Vilardaga et al., 2019, 2020).
3. *Let's Talk About Quitting Smoking* is a web-based intervention focused on Theory of Planned Behavior. It comprises 12 modules aimed to address attitudes, social norms, and perceived behavioral control for smoking and cessation treatment. It includes 48 interactive sessions that encourage the user to enlist reasons for smoking and track smoking related activities, practice coping skills, or learn about NRT and other medications, among others. The interface was designed to minimize reliance on cognitive functions to improve usability in patients with SMI (Brunette et al., 2019).
4. *Let's Talk About Smoking* is web-based motivational decision support system designed to increase motivation to quit. This program can be completed over a single sitting within 30–60 min. It includes a video program host who guides the participants through three modules to assist decision making about cessation treatment options. The program was tailored for people with cognitive deficits to ensure ease of use (Brunette et al., 2020).
5. *mSMART MIND* is a smartphone-assisted intervention based on mindfulness and CM. The intervention encourages mindfulness practices, incorporates EMA and uses a progressive mCM payment schedule through videos with a handheld CO monitor showing expired CO levels which are uploaded until 14 days after the set quit date (Minami et al., 2018).
6. *QuitGuide*, *QuitPal*, and *quitStart* are smartphone-based apps developed by the US National Cancer Institute based on different clinical practice guidelines for smoking cessation. *QuitGuide* is aimed toward the adult population, the app monitors behaviors and mood associated with quitting smoking, enhances motivation to maintain abstinence and helps patients setting a quit date. The intervention includes psychoeducation, tracking smoking habits and tips for quitting. *QuitPal* allows setting a quit date, daily tracking of smoking and mood, and allows users to log smoke-free days if the individual has been smoke-free for an entire day. The app allows for customized reminders, saving goals, and provides graphs of the progress (Vilardaga et al., 2016). *quitStart* was designed to target the teenage population. It encourages the user to set a quit date, provides education on smoking cessation strategies with interactive swipable cards, and allows for daily tracking of information. Additionally, it offers distraction games to aid with smoking craving. The app automatically sends check-in notifications to ask the user for the number of cigarettes smoked since the last check-in.
7. *WebQuit Plus* is a web-based intervention based on ACT, built on the *WebQuit Plus* program (Bricker, Mull, McClure, Watson, & Heffner, 2018). This program has four sequential components to help the user by making a quit plan, developing awareness and coping skills of triggers, and supporting long-term abstinence by engaging personal values and self-compassion. *WebQuit Plus* adds to the ACT program exercises to tackle specific challenges to smoking cessation, testimonials, and one- and two-way messaging to promote adherence to NRT and assistance with mood-specific triggers. It also uses tracking data to display money and minutes saved via reducing or quitting smoking on the main dashboard (Heffner et al., 2020).

**Table 2.** Effectiveness, acceptability, adherence, usability and safety of smoking cessation digital interventions

Digital intervention	Intervention studies	Format and availability online	Theoretical approach and duration	Effectiveness		Acceptability and adherence	Usability		Safety	
				Measure	Results		Measure	Results, mean (s.d.)		
iCOMMIT	Medenblik et al. (2020), Wilson et al. (2019)	Smartphone App Available: Partially available	Contingency management 6 weeks	Abstinence (self-reported 7-day PPA)	Development studies with successive cohort design reported abstinence rates of 38–40%. During the pilot trial, abstinence rates ranged from 9.5% (EOT) to 19.5% (EOT + 3 months). Although outcomes better than the control group (7.7 and 15.4%, respectively), *p* values were not reported.	High (37.4/40), but not statistically different from comparator (*p* = 0.91); 40–63% of patients showed high treatment adherence	NR	NR	NR	
Learn to Quit	Vilardaga et al. (2019, 2020)	Smartphone App Available: No	NR 2 weeks	Abstinence (self-reported 30-day PPA)	12.1% (no statistically significant differences with control group)	NR	SUS	83.1 (8.5)–85.2 (15.5); one article reports a statistically significant difference in usability respect to comparator (Cohen's *d* = 0.53, *p* = 0.046)	Reported in 2/2 articles; no statistically significant differences in AE compared to control group; no SAE reported; 1 participant hospitalized due to an unrelated psychiatric event	
				Attempt to quit	5.3 attempts					
Let's Talk About Quitting Smoking	Brunette et al. (2019)	Web-based Available: No	Theory of planned behavior 12 sessions	Abstinence (biochemically verified)	10%^a^	85% of participants reported a positive response to ‘What do you think about this intervention?’	Number of sessions accessed (total = 48)	23.6 (17.1)^a^	One patient stopped intervention because of increased paranoia	
				Cigarette smoking reduction (self-report)	25%^a^					
Let's Talk About Smoking	Brunette et al. (2011), Brunette et al. (2018a), Brunette et al. (2020)	Web-based Available: No	Theory of planned behavior Single session	Engagement in at least one smoking cessation intervention	67% (*p* < 0.05)^a^	NR 83.4% rated satisfaction with the intervention as ‘good or very good’^a^, and 3.3% as ‘hard to understand’^a^. Outcomes favored the intervention, although *p* values were not reported.	NR Perceived Usefulness and Ease of Use Scale	Intervention *v.* control, mean (s.d.) 8.9 (1.3) *v.* 8.3 (2.1), *p* = 0.045	No AE reported in 1/3 articles^8^	
				Abstinence and reduction of cigarettes smoked per day	Overall, no statistically significant differences were found with the placebo controlled and the no-ntervention groups^a^	Self-reported satisfaction rate was 95.4% (*v.* 83.1% in the control group)	Time spent using the intervention (total)^a^	58 min		
mSMART MIND	Minami et al. (2018), Minami et al. (2022)	Smartphone App Available: No	Contingency management + mindfulness 4 weeks	Abstinence (self-reported 7-day PPA at EOT)	12.5–40%	Intervention was found overall helpful (4.7–4.9/5)^9–10^ and enjoyable (4.6/5)^9^	Mindfulness practices per day	3–3.4	Reported in 1/2 articles; no AE found	
				Reduction in cigarettes/day	79.9–84%					
QuitGuide	Gowarty et al. (2021)	Smartphone App Available: Yes, free	Theory of planned behavior	Attempt to quit	86%^b^	NR	SUS	74 (7.6)	NR	
QuitPal	Vilardaga et al. (2016)	Smartphone App Available: No	Theory of planned behavior	NR	NR	NR	SUS	65.5 (18.6)	NR	
quitStart	Gowarty et al. (2021)	Smartphone App Available: Yes, free	Theory of planned behavior	Attempt to quit	86%^b^	NR	SUS	68 (22)	NR	
WebQuit Plus	Heffner et al. (2020)	Web-based Available: No	Acceptance and commitment therapy	Abstinence (self-reported 7-day PPA at EOT)	12% (not statistically significant from control group)	Only better than comparator in satisfaction and perceived usefulness	Number of logins, login days, days in use	No differences with comparator	18 AE reported: only 1 SAE was considered to have a potential relationship to the intervention; 3 AE were ‘possibly related’, and 2 AE ‘probably related’	

AE, adverse events; EOT, end-of-treatment; NR, not reported; PPA, point-prevalence abstinence; SAE, severe adverse events; SUS, System Usability Scale [mean (s.d.)].

a Results are reported for the whole group, not just people with severe mental illness (SMI).

b Results are reported jointly for *QuitGuide* and *quitStart*.

### Effectiveness

The definition of effectiveness varied widely among the different articles. The most frequently used definition was point-prevalent abstinence (PPA), either at 7 or 30 days, measured either as self-report or confirmed with expired CO. Abstinence rates ranged between 9 and 40%, with varying comparison groups. The highest abstinence rates were found for *mSMART MIND*, with biochemically verified 7-day PPA abstinence rates of 40% at 2 weeks after end of treatment (EOT), and 16% at 3 months post-EOT (Minami et al., 2022). Similar rates were found in *iCOMMIT*, with 7-day self-reported PPA at EOT of 38–40% for the successive cohort design (Wilson et al., 2019), but 9.5–19.0% for the pilot RCT trial (Medenblik et al., 2020). Other definitions of effectiveness used included reduction of cigarettes smoked per day (CPD), engagement in smoking cessation behavior, and smoking cessation knowledge. Vilardaga et al. (2020) found that *Learn to Quit* led to a significant greater reduction in CPD than the comparator (*QuitGuide*), and *iCOMMIT* was found to reduce CPD, although significant reductions were only reported for patients with BD. Regarding engagement in smoking cessation behavior (e.g.: seek counsel, start smoking cessation medication, enroll in support groups), the interventions did not seem to outperform their comparators. In this regard, Brunette et al. (2011); Brunette et al. (2018a) and Brunette et al. (2020) reported inconsistent evidence for the effect of *Let's Talk About Smoking*, and Heffner et al. (2020) and Vilardaga et al. (2020) did not find differences in NRT use between groups for *WebQuit Plus* and *Learn to Quit*, respectively. Finally, *iCOMMIT* was not found to improve smoking cessation knowledge (Medenblik et al., 2020). Detailed information regarding effectiveness can be found in Table 2.

### Acceptability and adherence

In the nascent field of digital health, the definitions of acceptability and adherence are oftentimes blurred and overlapped. For the purpose of this review, we report acceptability as the willingness and satisfaction to use a digital intervention (Nadal, Sas, & Doherty, 2020), and adherence as the degree to which patients are able to use the digital intervention as indicated by the research protocol.

Acceptability was reported only for three interventions (*Let's Talk About Quitting Smoking*, *Let's Talk About Smoking,* and *WebQuit Plus*). Across studies, acceptability was evaluated with author-developed scales rather than standardized measures. For *Let's Talk About Quitting Smoking* (Brunette et al., 2019), 87% of the sessions were rated ⩾3 on a 4-point Likert scale, and 85% of participants gave a positive response when interrogated about the intervention. Similarly, *Let's Talk About Smoking* was found to be easier to understand and to have a higher satisfaction rate than its comparator (Brunette et al., 2018a). For *mSMART MIND*, satisfaction was measured on a 5-point Likert scale, and the intervention was found ‘enjoyable’ (author-reported score: 4.6/5) and ‘helpful’ (author-reported score: 4.7–4.9/5) (Minami et al., 2018, 2022). *WebQuit Plus* outperformed its comparator (*Smokefree.gov*) in satisfaction and perceived usefulness, but not in other items (such as usefulness of the plans for quitting or the support forum, site organization or accessibility) (Heffner et al., 2020).

Additionally, three articles (Gowarty, Aschbrenner, & Brunette, 2021; Vilardaga et al., 2016, 2019) used semi-structured qualitative interviews to measure acceptability. Patients showed positive attitudes and high levels of acceptability for *QuitGuide* and *quitSTART*, emphasizing the motivational content (Gowarty et al., 2021). *QuitPal* was found to be excessively focused on quitting smoking, and that a shift toward reducing tobacco use would be preferable (Vilardaga et al., 2016). Finally, patients found *Learn to Quit* to be more engaging than *QuitGuide*, pointing out the use of gamification to boost engagement (Vilardaga et al., 2019). In general, the most remarkable features of the interventions were cigarette tracking, aids to avoid craving and monetary incentives (Gowarty et al., 2021; Vilardaga et al., 2016, 2019).

Adherence was reported for two interventions (*iCOMMIT*, and *mSMART MIND*). The intervention *iCOMMIT* was reported to have an adherence rate of 40–63% over the 4-week period of the trial (Wilson et al., 2019). Adherence, considered as percentage of videos uploaded as part of the mCM intervention, was reported to be 66.2–68.0% of the total requested videos (Minami et al., 2018, 2022). Relevant information regarding acceptability and adherence of the reviewed digital interventions can be found in Table 2.

### Usability

Five articles applied the System Usability Scale (SUS) to measure usability of four different digital interventions (Gowarty et al., 2021; Vilardaga et al., 2016, 2020, 2019). This 10-item scale provides a reliable measure of usability, with scores higher than 68 considered ‘above average’ (Brooke, 1996). The highest score was found for *Learn to Quit* (85.2 ± 15.5), while the lowest was reported for *QuitPal* (65.5 ± 18.6). In addition, Vilardaga et al., (2019) reported statistically significant higher SUS scores for *Learn to Quit* than its comparator (*QuitGuide*); and in a different article (Vilardaga et al., 2016), they found that patients with SMI found *QuitPal* challenging to use due to its layer structure. Heffner et al. (2020), found no differences between intervention and control groups for number of logins, number of unique login days or total number of days in use. Noteworthy, it was reported that none of the participants accessed the section where most of the content targeted to smokers with BD was located.

Additionally, three articles (Vilardaga et al., 2016, 2019; Wilson et al., 2019) used semi-structured qualitative interviews to measure usability. Using a think-aloud approach, users of *QuitPal* found obstacles to use several components of the app (e.g.: enter information, pull up the keypad, navigate through several layers), which ultimately required direct guidance (Vilardaga et al., 2016). *Learn to Quit* was found easier to use than *QuitGuide* (Vilardaga et al., 2019). Finally, Wilson et al. (2019) report that initiating CO readings was the main hurdle while using *iCOMMIT*. Relevant information regarding usability of the reviewed digital interventions can be found in Table 2.

### Safety

AEs were reported only for five digital interventions (*Learn to Quit*, *Let's Talk About Quitting Smoking*, *Let's Talk About Smoking*, *mSMART MIND*, and *WebQuit Plus*) in six articles (Brunette et al., 2019, 2020; Heffner et al., 2020; Minami et al., 2018; Vilardaga et al., 2020, 2019), although standardized assessments were not used. Most of the interventions were found to be safe, and no AEs were reported for *Let's Talk About Smoking* (Brunette et al., 2020) and *mSMART MIND* (Minami et al., 2018).

For *Learn to Quit*, Vilardaga et al. (Vilardaga et al., 2019, 2020) found no differences for ‘related’ or ‘possibly related’ AEs between intervention and control groups, with 87% of these AEs being linked to NRT use. Additionally, PANSS general psychopathology scores remained stable (pre- *v.* post-intervention: 40 *v.* 32, no *p* values reported), and although one participant was hospitalized due to an unrelated psychiatric event, no serious AEs (SAEs) were found. One participant complained of increased paranoia with *Let's Talk About Quitting Smoking* (Brunette et al., 2019). During the *WebQuit Plus* trial, Heffner et al. (2020) reported 18 AEs, of which nine were classified as SAEs. Five unique patients (two on the *WebQuit Plus* arm, and three on the control arm) reported seven SAEs that were characterized as psychiatric, but only one of these was deemed to have a potential relationship to the intervention. The remaining AEs were considered to be related to nicotine withdrawal symptoms or adverse effects of NRT use. In addition, depression and mania scores remained stable throughout the trial. Finally, Wilson et al. (2019) did not report any medication-related AEs with *iCOMMIT*, although no information was available for the digital component of this intervention. An overview of the safety of the interventions can be found on Table 2.

### Quality assessment

Overall, quality of the studies was ‘fair’ to ‘good’ as assessed with the NHLBI Study Quality Assessment Tools, although with limitations inherent to the experimental or pilot nature of most of the studies. Inter-rater agreement for quality assessment was 90.0% [95% CI (55.5–99.7)]. Studies showed high adherence to protocol and methods, with few drop-outs from the trials. However, blinding and sample size were found the main concerns when rating quality (see online Supplementary Tables S1 and S2 for detailed NHLBI assessments).

Digital interventions were evaluated using specific items of the A-MARS. In this regard, *quitStart* showed the greatest scores, mostly due to its highly user-friendly interface, with high ratings for engagement and functionality aspects. By contrast, *iCOMMIT* was deemed the lowest since the digital component of the intervention was mostly limited to mCM. Overall, the digital interventions showed good results in items of ‘interest’, ‘ease of use’ and ‘goals’, with lower ratings for addressing ‘multiple health issues/symptoms’ and ‘real-time tracking’. See online Supplementary Table S3 for detailed A-MARS ratings.

## Discussion

To our knowledge, this is the first systematic review of digital interventions for smoking cessation in patients with SMI. We found nine different digital interventions that were assessed in patients with SSD, BD, and MDD. First, the highest abstinence rates (measured as 7-day PPA) were found with *mSMART MIND* and *iCOMMIT*, two smartphone-based apps developed on the basis of mCM. In this regard, *mSMART MIND* seemed to be especially beneficial for patients with BD, showing a significant improvement in abstinence rates when compared with enhanced standard treatment without mCM (aOR = 8.12, 95% CI 1.42–46.6, *p* = 0.019). Additionally, *mSMART MIND* showed the greatest CPD reduction (79.4% at 2 weeks after EOT, decreasing to 61.4% at 3 months), followed by *iCOMMIT* and *Learn to Quit*. Results on motivation enhancement and improvement of smoking cessation knowledge do not support the use of the reviewed digital interventions for these purposes. Participants reported high acceptability rates for *Let's Talk About Quitting Smoking*, *Let's Talk About Smoking*, *iCOMMIT*, *mSMART MIND* and *WebQuit Plus*, and in the case of *Let's Talk About Smoking* and *WebQuit Plus*, acceptability measures outperformed those of the comparators. However, adherence rates to the interventions were low (40–68%). The highest usability score was found for *Learn to Quit* (SUS = 83.1–85.2%), which also was reported to be significantly better, in terms of usability, than *QuitGuide*. This seems in accordance with its user-centered design since the early phases of development, which allowed to tailor the intervention to patients with SMI. Results on qualitative measures were found to be similar to quantitative approaches, stressing the strength of using multiple methodologies when studying digital interventions. Finally, safety was reported for only five interventions, but broadly, the interventions were safe to use, and no psychopathological decompensations were reported while carrying out the trials. No AEs were reported for *Let's Talk About Smoking* or *mSMART MIND*, and in sum, only *WebQuit Plus* reported one psychiatric SAE that could potentially be related to the intervention.

In general, these findings suggest that digital interventions represent a suitable and promising alternative to promote smoking cessation in patients with SMI, although larger RCTs are clearly needed. Overall results are in line with similar reviews of digital interventions for smoking cessation in patients without SMI. Regmi, Kassim, Ahmad, and Tuah (2017) reported an improvement in quitting rates among smokers, although it was lower than in our findings (13–24%). This might be explained by the fact that in their review, most of the subjects were recruited through social media signs and advertisement. This could bias the recruitment: taking on less motivated subjects than the clinical populations included in the articles of our review may lessen quitting rates. In a 2019 update of their review, the Cochrane Tobacco Addiction Group found discouraging results for smoking cessation smartphone apps, reporting no evidence of benefit of these digital interventions for smoking cessation (Whittaker et al., 2019). Again, recruitment methods (online, through app stores) and the use of non-clinical populations might explain the discrepancies with our findings. The quality of the studies conducted on non-SMI population was deemed below average by the recent reviews on the matter (Chu et al., 2021; Regmi et al., 2017; Whittaker et al., 2019), and authors agreed on the need for more uniformity when conducting trials in this field. Using less stringent criteria due to the exploratory purpose of most of the included studies in our review, we found that the quality of the studies was fair to good, although more robust methodology is needed in order to be able to generalize these results and provide specific recommendations.

In the addiction field more broadly, some reviews have also found optimistic results on the use of digital medicine approaches for the treatment of methamphetamine (Rubenis, Baker, & Arunogiri, 2021) and alcohol (Colbert, Thornton, & Richmond, 2020) use disorders, albeit a nascent field, and methodological quality remains a significant barrier. Comparable results in terms of efficacy and acceptability were found in other areas of healthcare, such as cardiac rehabilitation (Wongvibulsin et al., 2021) or maternal health and pregnancy (DeNicola et al., 2020; Overdijkink et al., 2018), indicating the meaningful opportunity for digital medicine while highlighting the need for larger and methodologically comparable studies.

The results from this review should be interpreted with caution. Due to the different methodologies and ways of reporting effectiveness, acceptability, usability and safety, we are unable to provide a definite recommendation on which specific digital intervention should be used in clinical practice. Additionally, the relatively small number of participants in each study hinders the possibility of performing an adequately powered meta-analysis of the results. While *mSMART MIND* and *iCOMMIT* seem to be the most effective interventions in terms of abstinence, they are not available for public use in the most common operating systems, which deems its immediate application impossible. It is also important to note that although this review identified a total of nine digital smoking cessations trialed in SMI, only two of these (*QuitGuide* and *quitStart*) are readily available online, which showed poorer results in terms of effectiveness and have not been assessed as thoroughly as *mSMART MIND* and *iCOMMIT* for acceptability and safety. The general inaccessibility of evidence-based digital interventions for SMI is a problem which extends far beyond smoking cessation, with a recent review finding that only ~15% of apps trialing in schizophrenia are currently available online (Kwon, Firth, Joshi, & Torous, 2022). Clearly, further work is needed to improve the dissemination of effective digital approaches into the public sphere more generally.

In terms of effective strategies for smoking cessation in SMI, the results reported by both *mSMART MIND* and *iCOMMIT* appear to indicate that mCM could be the most effective approach. Nonetheless, future trials in this field should report standardized measures that allow a comprehensive comparison of effectiveness among different strategies, particularly because delivery of cash monetary reinforcement might be challenging in many settings (e.g.: public health systems, private practices), whereas a digitally delivered reinforcement may easily overcome such limitations. Moreover, results from the *Learn to Quit* and *WebQuit Plus* trials highlight the need for simplified interfaces to improve acceptability and usability in SMI. In this regard, the idiosyncrasies of patients with SSD, BD, and MDD must be considered, and research efforts targeted to each individual disorder must be developed. For instance, nicotine has been reported to affect neurocognitive performance in patients with schizophrenia, but not in patients with BD and MDD (Morisano et al., 2013). Also, patients with SSD exhibit impaired cognitive performance (Green, Kern, Braff, & Mintz, 2000; Mallet et al., 2022), which might impact their ability to use digital interventions. Considering this, patients with SSD might face harder cognitive challenges than patients with BD and MDD when quitting smoking, and thus interventions tailored to their ability might be necessary.

Another limitation when considering the results of this review is that all the digital interventions were studied in the US and in stable outpatients, which may limit the generizability of the results to other settings and patient populations. Given that most digital interventions were not readily available to be systematically evaluated with a standardized evaluation scale, results from our assessments should be cautiously considered. Further, reports of AEs in behavioral interventions were scarce and not systematically reported, in contrast with other types of clinical trials, which could lead to the misguided conclusion that non-pharmacological interventions are without risk. As described in previous literature, AEs are also present in both psychotherapeutic and behavioral interventions and should be systematically defined and reported to avoid harming in other patients (Linden & Schermuly-Haupt, 2014). Finally, concerns regarding cybersecurity should be monitored in the future to guarantee privacy and confidentiality to the users.

In conclusion, digital health appears to be a promising field to develop and further validate interventions for smoking cessation, which should also be tailored to patients with SMI. While some interventions have shown positive preliminary data, more research is needed to provide clinicians and patients with robust recommendations to treat tobacco use with digital interventions.

## Supplementary material

For supplementary material accompanying this paper visit https://doi.org/10.1017/S003329172300123X. click here to view supplementary material
