# Supplementary material for: A systematic review of digital interventions for smoking cessation in patients with serious mental illness
Source: Psychol Med. 2023 May 10;53(11):4856–68. doi: 10.1017/S003329172300123X (PMC10476065; doi:10.1017/S003329172300123X)
Supplement: Supplementary file 1 [file S003329172300123Xsup001.docx]

**Supplementary table 1.** Quality assessment of controlled intervention studies based on NHLBI Study Quality Assessment Tools for Controlled Interventions.

|  | Brunette, 2011 | Brunette, 2017 | Brunette, 2020 | Heffner, 2019 | Medenblik, 2020 | Vilardaga, 2019 | Minami, 2021 |
| --- | --- | --- | --- | --- | --- | --- | --- |
| Was the study described as a randomized, a randomized trial, a randomized clinical trial, or an RCT? | No | Yes | Yes | Yes | Yes | Yes | Yes |
| Was the method of randomization adequate? | Yes | Yes | Yes | NR | Yes | Yes | Yes |
| Was the treatment allocation concealed? | No | Yes | Yes | Yes | Yes | Yes | Yes |
| Were the study participants and providers blinded to treatment group assignment? | No | No | Yes | Yes | No | NR | No |
| Were the people assessing the outcomes blinded to the participants’ group assignments? | No | NR | Yes | Yes | No | No | No |
| Were the groups similar at baseline on important characteristics that could affect outcomes? | Yes | Yes | Yes | Yes | Yes | No | Yes |
| Was the overall drop-out rate from the study at endpoint ≤20% of the number allocated to treatment? | Yes | Yes | Yes | Yes | NR | Yes | Yes |
| Was the differential drop-out rate at endpoint ≤15%? | Yes | Yes | Yes | Yes | NR | Yes | Yes |
| Was there high adherence to the intervention protocols for each treatment group? | Yes | Yes | Yes | Yes | Yes | Yes | Yes |
| Were other interventions avoided or similar in the groups? | No | Yes | Yes | Yes | Yes | Yes | Yes |
| Were outcomes assessed using valid and reliable measures, implemented consistently across all study participants? | No | Yes | Yes | Yes | Yes | Yes | Yes |
| Did the authors report that the sample size was sufficiently large to be able to detect a difference in the main outcome between groups with at least 80% power? | No | No | No | NA | NA | NR | No |
| Were the outcomes reported or subgroups analyzed prespecified? | No | NA | No | NA | Yes | Yes | No |
| Were all randomized participants analyzed in the group to which they were originally assigned? | Yes | Yes | Yes | Yes | Yes | Yes | Yes |
| Quality rating | Fair | Good | Good | Good | Good | Good | Fair |

Abbreviations: NA: not applicable; NHLBI: National Heart, Lung, and Blood Institute; NR: not reported, RCT: randomized clinical trial.

**Supplementary table 2.** Quality assessment of before-after (pre-post) studies with no control group based on NHLBI Study Quality Assessment Tools for Before-After (Pre-Post) Studies With No Control Group.

|  | Brunette, 2019 | Minami, 2017 | Wilson, 2018 |
| --- | --- | --- | --- |
| Was the study question or objective clearly stated? | Yes | Yes | Yes |
| Were eligibility/selection criteria for the study population prespecified and clearly described? | No | Yes | Yes |
| Were the participants in the study representative of those who would be eligible for the test/service/intervention in the general or clinical population of interest? | Yes | No | Yes |
| Were all eligible participants that met the prespecified entry criteria enrolled? | Yes | Yes | Yes |
| Was the sample size sufficiently large to provide confidence in the findings? | No | No | No |
| Was the test/service/intervention clearly described and delivered consistently across the study population? | Yes | Yes | Yes |
| Were the outcome measures prespecified, clearly defined, valid, reliable, and assessed consistently across all study participants? | No | No | Yes |
| Were the people assessing the outcomes blinded to the participants’ exposures/interventions? | No | Yes | No |
| Was the loss to follow-up after baseline ≤20%? Were those lost to follow-up accounted for in the analysis? | Yes | No | Yes |
| Did the statistical methods examine changes in outcome measures from before to after the intervention? Were statistical tests done that provided p values for the pre-to-post changes? | Yes | No | No |
| Were the outcome measures of interest taken multiple times before the intervention and multiple times after the intervention? | No | No | No |
| If the intervention was conducted at a group level, did the statistical analysis take into account the use of individual-level data to determine effects at the group level? | NA | NA | NA |
| Quality rating | Fair | Fair | Fair |

Abbreviations: NA: not applicable; NHLBI: National Heart, Lung, and Blood Institute.

**Supplementary table 3.** Digital intervention quality ratings based on A-MARS.

|  | Engagement | Interest | Customization | Interactivity | Ease of use | Goals | Evidence base | Strategies | Solutions | Multiple health issues/symptoms | Real time tracking | Total |
| --- | --- | --- | --- | --- | --- | --- | --- | --- | --- | --- | --- | --- |
| *iCOMMIT* | 3 | 2 | 1 | 2 | 3 | 4 | 2 | 1 | 1 | 1 | 1 | 21 |
| *Learn To Quit* | 5 | 5 | 3 | 4 | 4 | 4 | 3 | 4 | 3 | 3 | 2 | 40 |
| *Let’s Talk About Quitting Smoking* | 3 | 5 | 3 | 4 | 4 | 4 | 3 | 4 | 3 | 2 | 1 | 36 |
| *Let’s Talk About Smoking* | 4 | 4 | 3 | 3 | 4 | 4 | 2 | 3 | 3 | 2 | 1 | 33 |
| *mSMART MIND* | 4 | 4 | 3 | 5 | 5 | 5 | 3 | 4 | 4 | 3 | 1 | 41 |
| *QuitGuide* | 4 | 4 | 4 | 4 | 5 | 4 | 3 | 3 | 3 | 2 | 5 | 41 |
| *QuitPal* | 3 | 3 | 3 | 3 | 3 | 3 | 2 | 3 | 2 | 2 | 1 | 28 |
| *quitStart* | 5 | 5 | 5 | 5 | 4 | 4 | 3 | 5 | 4 | 3 | 5 | 48 |
| *WebQuit Plus* | 4 | 4 | 4 | 5 | 4 | 4 | 3 | 3 | 3 | 4 | 5 | 43 |
